# A Review of Infrared Thermography for Delamination Detection on Infrastructures and Buildings

**DOI:** 10.3390/s22020423

**Published:** 2022-01-06

**Authors:** Ko Tomita, Michael Yit Lin Chew

**Affiliations:** Department of the Built Environment, National University of Singapore, Singapore 117566, Singapore; bdgchewm@nus.edu.sg

**Keywords:** infrared thermography, delamination, building, infrastructure, time window, environment, infrared camera, target object, thermal property

## Abstract

This paper provides a comprehensive review on the use of infrared thermography to detect delamination on infrastructures and buildings. Approximately 200 pieces of relevant literature were evaluated, and their findings were summarized. The factors affecting the accuracy and detectability of infrared thermography were consolidated and discussed. Necessary measures to effectively capture latent defects at the early stage of delamination before crack formation were investigated. The results of this study could be used as the benchmarks for setting standardized testing criteria as well as for comparison of results for future works on the use of infrared thermography for detection of delamination on infrastructures and buildings.

## 1. Introduction

With the aging of civil infrastructures and buildings, those deterioration has become an important social issue that can threaten public safety. The American Road & Transportation Builders Association reported in 2020 that 36% of bridges in the US need replacement or rehabilitation due to their aging [1]. Similarly, in Singapore, the age of 74% of high-rise residences exceeds 20 years old, and more than 90 incidents of falling parts of facades from high places occurred in recent three years [2]. To ensure public safety, governments introduced mandatory periodic inspection schemes of infrastructures and buildings. For civil infrastructures, long highway bridges in the US are required to be inspected every 24 months [3]. For buildings, Singapore [2], Japan [4], Hong Kong [5], and 13 cities in the US and Canada [6] enacted periodic inspection laws to prevent falling objects from building facades.

Defects in infrastructures can be diverse and include delamination, cracks, staining, and spalling, caused mainly by water penetration, reinforcement corrosion, thermal/moisture movements, differential settlement/loading, poor construction practices, etc. [7,8]. Among them, delamination, the condition in which the surface and inside are unbonded or unintegrated properly, are crucial because they lead to further deterioration, such as crack formation and element falling [9]. In concrete infrastructures, delamination arises in concrete cover near the surface because of the expansion of corroded embedded rebars as well as cyclical traffic load stress and environmental changes [10]. In building facades, delamination generally occurs at the interface between a finish layer, such as tiles or render, and a substrate, such as concrete or bricks [8,11,12,13]. Delamination constitutes a significant part of defects occurring on tile façades, accounting for 27% of facade defects in Singapore [8] and 71% in Brazil [14]. Since delamination arises under the surface, it is to be detected via nondestructive testings (NDTs).

In recent decades, various NDTs were developed to detect defects in multiple fields since they can evaluate object characteristics [15,16,17]. Each NDT has different principles and features, so that it is necessary to select appropriate NDTs according to the purpose and conditions of inspection [18]. Several NDTs can identify delamination, e.g., tapping tests, chain drag tests, hammer sounding tests, ground-penetrating radar, and infrared thermography (IRT) [19,20]. Among them, IRT especially drew increasing attention due to its advantages of real-time [21], contactless [22], and wide-area measurements [23]. Another advantage is that the price of an infrared (IR) camera has recently become affordable [21]. Therefore, IRT can serve as a suitable NDT for civil infrastructures and buildings.

IRT is defined as a process of measuring surface temperature distribution using IR cameras and processing and interpreting the data of IR images [24]. For infrastructures and buildings, IRT is used not only for delamination detection but also a wide range of inspections: moisture [25,26,27,28,29,30,31], thermal insulation [7,32,33,34,35], internal structure [36], cracks [37,38,39], air leakage [40,41], and cultural heritage [42,43,44,45,46]. In terms of delamination on infrastructures and buildings, IRT generally employs a passive analysis scheme, which uses surrounding environments as heat sources to stimulate temperature distribution [7,47,48]. However, passive IRT has some limitations at the step of data acquisition. The most critical limitation is that the detectability of passive IRT depends on uncontrollable environmental conditions, such as solar irradiation, ambient temperature, and wind [49,50,51,52]. Even in the same infrastructure, microclimates around surfaces differ depending on surface directions [53]. Other factors that may affect the detectability include delamination properties, target object [54], and IR camera [52]. If IR images are measured without due consideration of these conditions, delamination may be overlooked or misinterpreted. Understanding the mentioned conditions is hence crucial for planning and conducting passive IRT. Thus, many studies were conducted on the effects of environmental conditions, delamination properties, target objects, and IR cameras [55]. However, inconsistent results were often observed because of different conditions of experiments [56].

This paper focuses on the use of IRT to detect delamination on infrastructures and buildings to prevent falling objects from heights that endanger public safety. It provides a comprehensive review of the use of IRT by providing backgrounds, principles, and state-of-the-art knowledge on affecting factors and desirable conditions. This paper will contribute to increasing the reliability of IRT and facilitating further research.

Section 2 of this paper presents related review papers of IRT inspection on infrastructures and buildings. Section 3 explains the theory of temperature measurement and classifications of IRT. In Section 4, the principle and analysis methods of IRT for delamination detection and existing standards and guidelines are described. Additionally, the performance of IRT in detecting delamination is compared with that of other NDTs. Section 5 compiles and discusses some of the latest case studies on the impact of the various factors and investigates the different methodologies adopted. Section 6 compares and synthesizes relevant literature on factors affecting detectability. Lastly, Section 7 states conclusions.

## 2. Related Review Works on IRT

This section investigates review papers on IRT within the last decade. Recent review papers on IRT were conducted from perspectives of applications, methodologies, and research trends.

The first perspective is IRT applications, which are commonly used in reviews. Application reviews range from the level of introducing case studies in industrial fields to the level of in-depth investigation of a specific application. IRT applications were developed in many fields, including medical [57], aerospace [58], plant [59], electronic component [52,60], gas [61], machine [57,62], metal corrosion [63], photovoltaic panels [64], composite materials [65,66,67], and cultural heritage [68,69]. Similarly, various IRT applications were proposed for infrastructure and building inspection. Garrido et al. [22] introduced past studies in terms of inspected subjects: buildings, civil infrastructures, and heritage sites. Among these types, applications for civil infrastructures and buildings are the main subject of review papers.

Several review papers focused on the energy audit of building envelopes using IRT to evaluate building energy performances [7,33,48,50,70]. Lucchi [70] reviewed detailed applications of energy audit: detection of thermal bridge, insulation defects, air leakage, and moisture; indoor temperature and U-value measurements; and human comfort assessment. Among those applications, Nardi et al. [71] focused on quantitative IRT for the U-value measurement of building walls, representing building energy efficiency. Similarly, Bienvenido–Huertas et al. [72] focused on IRT as one of the in-site methods for assessing U-value. Apart from energy audits, another important application of IRT in civil infrastructures and buildings is the detection of delamination, void, and high moisture content. Lourenço et al. [9] investigated state-of-the-art techniques of IRT to detect delamination and moisture beneath ceramic claddings facades to reveal efficient quantitative and qualitative survey methods. Meanwhile, Sirca Jr. & Adeli [73] focused on experiment conditions and examined previous studies on IRT for concrete defect detection in laboratory tests and in field surveys. These reviews indicate the effectiveness of IRT to detect delamination on infrastructures and buildings.

The second perspective is the methodologies of conducting IRT. A wide variety of methodologies was developed and is classified based on their features and principles [63]. One classification of IRT is an analysis scheme including passive IRT, active pulsed IRT, and active lock-in IR [21]. Among them, Milovanović & Pečur [51] focused on active IRT for concrete infrastructures and described physical backgrounds, equipment, and postprocessing methods. Furthermore, Garrido and coauthors reviewed IRT methodologies for infrastructures during data acquisition [55] and postprocessing [74], respectively. During the data acquisition step, the authors introduced the IRT approaches for data collection and compared the latest studies regarding experimental setups, target materials, IRT modes, and analysis schemes in each defect type and application [55]. At the postprocessing step, the authors introduced the theories and representative studies on analysis algorithms and discussed those advantages and disadvantages. These reviews provide overviews of traditional and latest IRT methodologies.

The final perspective is the analysis of research trends based on statistical data of the number of past studies. For example, Fox et al. [75] analyzed research trends about IRT of energy-related building defects detection and discussed the correlation between the types of methodologies. Similarly, Kylili et al. [21] statistically analyzed research trends of IRT in building facades regarding measurement methods, analysis schemes, and analysis types. These statistical reviews objectively indicate the increase of literature on IRT for infrastructures and buildings.

As mentioned above, previous reviews about IRT were conducted from various perspectives. However, no review focuses on the characteristics of detectable delamination and measurement conditions affecting reliabilities and detectability of IRT for infrastructures and buildings.

## 3. Infrared Thermography

### 3.1. Theory of Temperature Measurement

This section explains the principle of temperature measurement by IR cameras. Heat energy can be transferred in three ways: conduction, convection, and radiation. Temperature measurement by IR cameras utilizes radiation transfer. All objects with an absolute temperature greater than 0 K emit electromagnetic waves, mainly in infrared spectra. According to Stefan–Boltzmann’s law, radiant energy from a black body is as follows:

*W_b_* = *σT_obj_*^4^ (W/m^2^), (1)

where *W_b_* is the total radiant flux emitted per unit area (W/m^2^), *σ* is the Stephan–Boltzmann constant, and *T_obj_* is the absolute temperature of the object (K). The black body is defined as an ideal object that absorbs all the radiation that collides with it at any wavelength. However, an actual object, called a grey body, is not a black body because objects have some reflection and transmission. The radiant energy emitted by a grey body (*W_g_*) is as follows:

*W_g_* = *εW_b_* = *εσT_obj_*^4^ (W/m^2^), (2)

where *ε* is emissivity, defined as the ratio of the radiant energy emitted from the object to the energy emitted from the black body at the same temperature.

**Figure 1:**
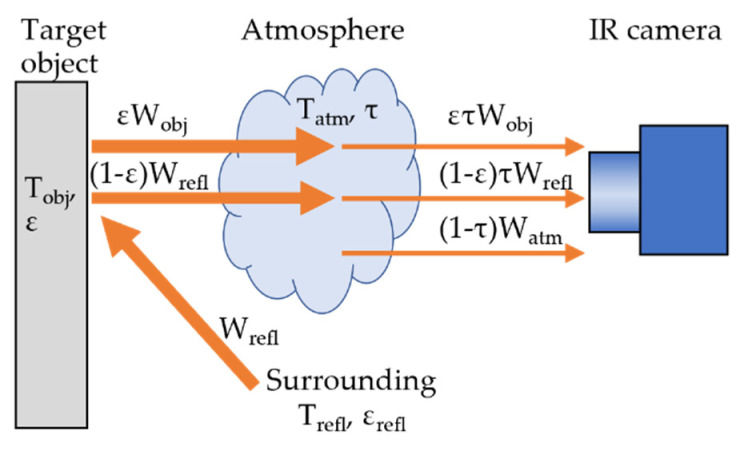
Diagram of temperature measurement by infrared (IR) camera.

IR cameras capture radiant energy in a specific infrared spectrum region emitted from an object and convert the energy into a temperature value. Figure 1 shows the elements of thermal radiation captured by an IR camera when measuring the surface temperature of an opaque object. Infrared radiation received by an IR sensor consists of three sources: emission from the target object (*W_obj_*), assuming the object as the black body, emission from surroundings reflected on the object (*W_refl_*), and emission from the atmosphere (*W_atm_*). The following formula expresses the total heat radiation detected by the IR camera (*W_total_*):

*W_total_* = *ετW_obj_* + (1 − *ε*)*τW_refl_* + (1 − *τ*)*W_atm_*, (3)

where *τ* is the transmittance of the atmosphere. The reflected radiation assumes that reflection temperature *T_refl_* is the same for all reflections from surroundings, and the emissivity of surrounding surfaces *ε_refl_* is assumed to be one. Radiation from the object and reflected radiation are absorbed by the atmosphere during traveling. Atmospheric radiation is emission from the atmosphere between the object and the camera at ambient temperature *T_atm_*. “1 − *τ*” indicates the atmosphere’s emissivity, and *τ* depends on *T_atm_*, relative humidity, and measurement distance between the target object and the IR camera. Therefore, accurate surface temperature measurements need to be compensated for the effects of emissivity *ε*, ambient temperature *T_atm_*, relative humidity, reflection temperature *T_refl_*, and distance [76].

In particular, the emissivity of target objects has a significant influence on temperature measurement [77]. The emissivity is a value from 0 to 1 and varies depending on materials, surface texture, angle, wavelength, and surface temperature [78]. Materials generally used in infrastructure, such as concrete, plaster, and general paint, have a high emissivity of 0.70–0.95 [78,79]. Thus, qualitative evaluation of defects can use emissivity values listed in emissivity libraries, whereas accurate temperature measurements for quantitative evaluation require the measurement of emissivity of target materials [50].

### 3.2. Classification of IRT

There are various methodologies of conducting IRT. They are classified from several viewpoints: analysis scheme, mode, and measurement method [50]. When assessing defects or thermal performances on infrastructures and buildings, it is crucial to select appropriate methodologies based on the purpose of the survey and conditions.

#### 3.2.1. Analysis Scheme

An analysis scheme is a classification criterion based on the origin of the energy input to a target object to generate temperature distribution on the surface. There are passive and active IRT.

Passive IRT utilizes natural heat sources as stimuli to generate a thermal gradient inside an object, causing thermal contrast on the object’s surface between sound and defect area [50]. The primary heat sources are generally solar irradiation and ambient temperature [22]. The natural heat sources heat large areas uniformly, so that passive IRT can inspect an extensive infrastructure at one time. Additionally, passive IRT does not require artificial heat sources, resulting in low cost. However, the detectability and accuracy of passive IRT significantly rely on various factors such as weather, surface orientation, and sunlight direction [50], so that the detectability of passive IRT may be limited. Additionally, passive IRT is not suitable for quantitative evaluation because the natural heat sources cannot be controlled. Therefore, passive IRT is mainly applicable for identifying defect locations before conducting advanced NDTs and is often used to inspect civil infrastructures and building facades [50,51,75].

Active IRT uses artificial heat sources to heat a target object to generate a thermal gradient [51]. Active IRT typically captures clear visualization of thermal anomalies compared to passive IRT [80] and can survey under conditions difficult for passive IRT. Traditional artificial heat sources are heat guns and hot water jets and bags [55]. Advanced thermal excitations include thermal induction, laser, ultrasonic, and microwave [22,81]. The mainstream for infrastructure inspection is optical excitation, such as halogen lamps and xenon lamps. Furthermore, controlled energy input allows quantitative assessment of defects, for example, defect depth [21]. Thus, active IRT is suitable for investigating specific areas in detail, such as heritage sites [75]. However, artificial heat sources are difficult to heat large areas uniformly, so that active IRT is not suitable for surveying large areas, such as buildings and infrastructures.

Active IRT is further classified according to heating processes: pulsed IRT (PT), step heating thermography (SH), and lock-in IRT (LT) [9]. PT provides a short pulse thermal stimulus of milliseconds and analyzes decreasing temperature curves [22]. SH is a method of applying a long-term thermal excitation pulse, called long-pulsed IRT, square-pulse IRT, or conventional IRT [9,55,82]. LT supplies a modulated sinusoidal wave energy, synchronizes an IR camera with energy input, and measures its thermal response’s phase difference and amplitude [55]. Laboratory tests or field surveys for infrastructures by active IRT usually adapt SH. This is probably because common construction materials, such as concrete, have lower thermal diffusivity than metals, hence a long heating time is required to cause thermal response [82].

#### 3.2.2. Mode

IRT is also classified into two modes according to the relative position of an IR camera and a heat source: transmission mode and reflection mode [55,66]. These modes require different environmental conditions for measurement.

The transmission mode places a heat source on one side and an IR camera on the opposite side of a target object [66]. The temperature difference between both surfaces generates heat flow passing through the target object. Defect areas have different thermal properties from sound areas and disturb the heat flow, leading to nonuniform thermal distribution on the opposite surface. Thus, this mode can detect deep defects and internal structure differences, so that it is commonly used for energy audits to diagnose insulation defects, moisture, and air leakage [48,55]. Additionally, this mode can quantitatively evaluate the U-value based on heat flux and the temperature difference between both surfaces [83]. However, The transmission mode needs to access both sides of the object. Moreover, as heat flow may take a long time to pass through an object, transmission mode requires keeping the difference between the inside and outside temperature for a long period to achieve a thermal equilibrium state in walls [7,33,48,83]. For instance, the British standard [7] states test requirements of a stable ambient temperature for at least 24 h before the measurement and no exposure to direct sunlight for at least 12 h. Therefore, thin building walls are appropriate for this mode.

The reflection mode places a heat source and an IR camera on the same side of a target object [66]. In this mode, radiation detected by the camera comes from heat flow reflected by defects [55]. Thus, this mode is suitable for detecting subsurface delamination at shallow places [55]. The advantage of the mode is that IRT can be conducted with access to only one side of the object. Therefore, the reflection mode is often applied to delamination inspection for infrastructures and buildings. However, it demands dynamic energy input into the surface from the outside by radiation or convection [51].

#### 3.2.3. Measurement Method

IRT has two measurement methods: qualitative and quantitative IRT [50]. Qualitative IRT evaluates defects from color patterns indicating temperature in IR images [75]. Qualitative IRT does not require measuring accurate temperature values [22]. The primary aim of the survey for infrastructures is generally the investigation of the presence and location of defects. Thus, qualitative IRT is commonly employed as standards and guidelines [7,26,33,47,48,79] due to its simplicity. However, it is not easy to provide information on defect properties or severity levels [50].

Quantitative IRT is a numerical evaluation method by comparing temperature values on IR image pixels between identical items or baselines [50]. The quantitative method can assess defect properties or levels of severity. Various quantitative methods were studied, for example, the thermal resistance of walls [84], depth of delamination [85], and moisture content in lightweight concrete [86]. The challenge of this IRT is the requirement to measure accurate temperature. Hence, IR images need to be compensated for emissivity, atmospheric attenuation, and reflected temperature [22], in addition to the thermal properties of the tested object [50].

## 4. Delamination Detection

### 4.1. Principle of Delamination Detection

**Figure 2:**
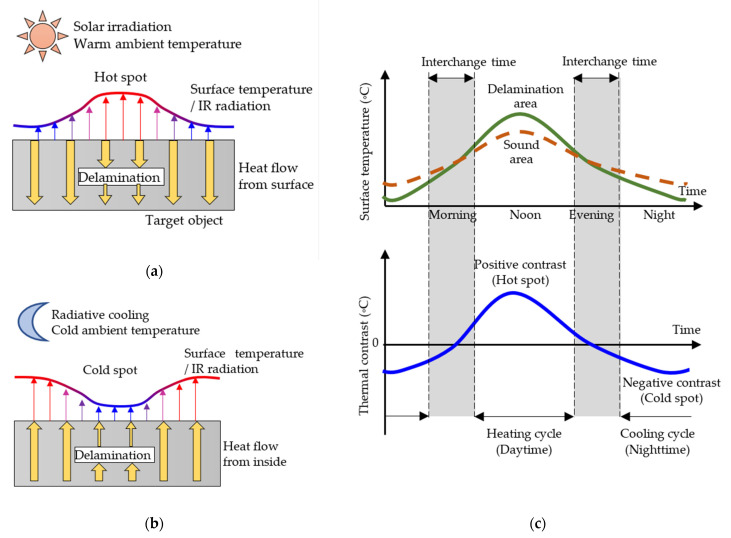
Principle of thermal contrast generation due to delamination: (**a**) diagram of heat flow during the heating cycle (daytime); (**b**) diagram of heat flow during the cooling cycle (nighttime); (**c**) daily changes of surface temperatures and thermal contrast.

IRT for detecting subsurface delamination on infrastructures and buildings generally adopts passive IRT of the reflection mode [47]. The principle of passive IRT is capturing thermal contrast between delamination area and sound area due to nonuniform heat flow [87]. Figure 2 shows the heat transfer in a target object with delamination during (a) a heating cycle and (b) a cooling cycle. Figure 2c illustrates typical daily changes of surface temperature and thermal contrast on a sunny day.

**Figure 3:**
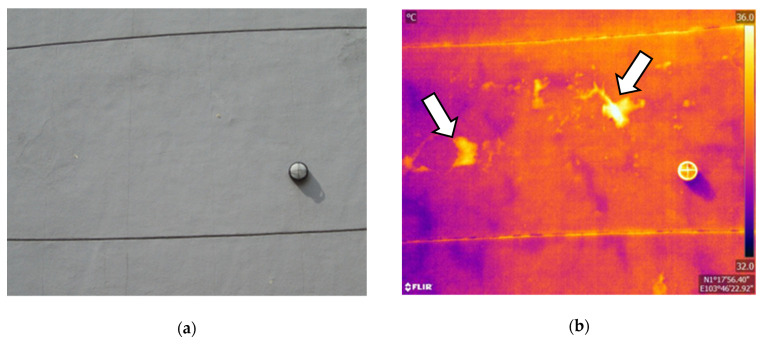
An example showing images of delamination on a building wall during heating cycle: (**a**) visual image; (**b**) IR image. Arrows indicate delamination areas.

From early morning to noon, solar radiation increases, and the ambient temperature rises. Solar irradiation and warm ambient temperature heat the surface of a target object, creating heat flow to the inside of the object. The thermal conductivity of concrete is approximately 1.6 W/mK, while that of air, filling delamination, is significantly low at 0.024 W/mK [88]. Thus, delamination acts as insulation and disturbs heat flow. As a result, the surface temperature above delamination becomes higher than the temperature of the surrounding area. Delamination areas appear as positive thermal contrast or hot spots in IR images, as shown in Figure 3. This period during daytime is called a heating cycle [89].

On the other hand, during nighttime, the surface temperature declines due to radiative cooling and low ambient temperature [90]. The heat energy stored in a target object during daytime transmits toward the surface, while this heat flow is obstructed by delamination. As a result, the surface temperature above delamination becomes lower than that of the surroundings. Delamination appears as negative thermal contrast or a cold spot. This period during nighttime is called a cooling cycle [89].

IRT survey can be conducted during both the heating cycle and the cooling cycle. However, the periods when the two cycles exchange in the early morning and the evening, called interchange times [90], are not recommended for IRT surveys. The reason is that interchange time has lower thermal contrast than the two cycles, as shown in Figure 2c.

### 4.2. Analysis Method

Analysis methods for delamination detection from IR images were developed as postprocessing procedures [74]. The analysis methods are divided into two groups based on the number of IR images used for analysis: one-time data analysis and time-series data analysis.

#### 4.2.1. One-Time Data Analysis

One-time data analysis, called single-frame image processing [91], processes a single IR image at a specific moment. This analysis requires only one captured IR image of target objects, so that an inspector can survey large areas efficiently with one IR camera. Thus, the analysis is widely used for infrastructure and building surveys [47,92,93]. However, the analysis tends to be subject to noise due to surroundings and nonuniform heatings [94]. The one-time data analysis includes (a) visual evaluation, (b) thermal contrast, and (c) image processing.

(a) Visual Evaluation

Visual evaluation is a method that an inspector interprets temperature distribution patterns in an IR image by comparing surroundings and assesses the presence and location of delamination [95,96,97]. This evaluation is practical and has also been adopted in surveys [7,48]. One problem is that its accuracy and detectability depend on the inspector’s experience, intuition, and judgment [98]. Therefore, interpretation should be conducted by a qualified inspector of IRT [99] to ensure inspection qualities. Another problem is that color scales representing temperature values need to be set in proper temperature ranges to avoid overlooking delamination [95,100,101,102]. For example, Washer et al. [100] suggested the range of 2.2–4.4 °C for shaded areas.

(b) Thermal Contrast

Thermal contrast, called ΔT, is referred to the surface temperature difference between the delamination area and the sound area [88,100]. Thermal contrast is a primary quantitative indicator to evaluate delamination in previous studies [10,88,103]. Thermal contrast may be due to causes other than delamination, such as surface conditions, subsurface materials, or object shape.

(c) Image Processing

Image processing was developed to extract temperature abnormalities automatically, quantitatively, accurately, and sensitively. The processing mainly utilizes threshold temperature values and temperature gradients.

Threshold temperature values are generally used for image processing. This image processing sets a threshold temperature value to judge areas as delamination and converts an IR image into a binary image based on the value. The processing has the advantages of simple evaluation; however, this processing primarily has two challenges.

The first challenge is determining threshold values because the values are affected by environmental conditions. Therefore, various methods to decide threshold values were proposed. The primitive method is that an inspector decides a threshold value that gives clear contrasts between sound and delamination areas by changing the value. The disadvantages of this method are subjective and time-consuming [104]. Japanese guideline of tile façade inspection [96] proposed that a delamination area is confirmed by the tapping method in advance, and the temperature difference between the delamination area and surrounding area is used as the value. Another approach is analyzing the temperature histogram of an IR image to determine the threshold value objectively. Garrido et al. [105] assumed that the histogram was a bimodal distribution composed of sound and delamination area. They acquired the temperature of the modal overlapping point by the Otus method as the threshold value. Meanwhile, Omar et al. [106] employed a k-means clustering method, an unsupervised machine learning method, to divide temperature values in an IR image into multiple clusters. They considered the boundary temperature values of clusters as the threshold values.

The second challenge of threshold values is difficult to evaluate the entire target object by one global threshold value. The reason may be that the entire surfaces of infrastructures or buildings are not under the same conditions, and each local area has a different average temperature and gradient [104]. Thus, methods for detecting temperature anomalies in local areas rather than in a global area were proposed. For example, Oh et al. [104] simply divided the IR image of a bridge deck into 16 local areas and used different threshold values for each area. Park et al. [107] extracted wall areas from building facades in visual images using a convolutional neural network (CNN) and analyzed the threshold values within wall areas. Cheng et al. [108] developed a delamination segmentation technique that extracts regional maximum temperature by a weight decay function. In these ways, it is necessary to limit the region of interest by some methods.

Temperature gradients are also employed for image processing. The processing identifies the areas of thermal anomalies based on the significant temperature changes at the edge of delamination. The advantages of the gradient are that measuring accurate temperature values is not required [109], and a slight temperature gradient over the entire surface may not be judged as delamination. For example, Lia et al. [109] identified delamination areas precisely by a spatial pixel differentiation algorithm even under unfavorable measurement conditions. In addition, Cheng & Shen [110] proposed temperature gradient-based level set method (LSM) and showed that LSM was more accurate and stable detection than the k-means method.

Overall, A substantial number of image processing methods using one IR image were developed. However, they may be designed to be optimized under specific conditions. To improve the accuracy of detectability and applicability for field inspection, further research is needed.

#### 4.2.2. Time-Series Data Analysis

Time-series data analysis collects courteous IR images over time and analyses time-series temperature data. It is also called time-lapsed thermography [36,75], time-dependent IRT [9], or continuous multiframe image processing [91]. The advantages of this analysis are robust to noise by nonuniform environment conditions [94] and high detectability [82]. It also allows conducting the quantitative assessment of delamination depth [85]. Thus, various image processing methods using multi-IR images were developed [22,74,91]. For example, simple image subtraction (SIS), also known as the computation of image differences, subtracts temperatures between two IR images at the same pixel location [111]. Principal component thermography (PCT), advanced processing based on principal component analysis (PCA) to summarize high-dimensional data [22], transforms a temperature 3D matrix in a combination of space and time into a 2D matrix by singular value decomposition to extract features and reduce noise [112,113]. Pulsed phase thermography (PPT), a method based on active IRT with one-dimension discrete Fourier transform, converts time-domain temperature data into frequency-domain data [112]. PPT has the advantage of suppressing the effects of spatially nonuniform heating and emissivity distribution [114]. Additionally, Cotič et al. [82] stated that PPT increased the maximum detectable depth by 50% over thermal contrast of one-time data analysis. In addition to the above, other methods were proposed including nonnegative matrix factorization (NMF) [111,115] and wavelet transformation [116]. Although time-series data analysis tends to be superior to one-time data analysis about detectability, the analysis requires fixing IR cameras and measuring the same object for a long duration. Therefore, time-series analysis is suitable for detailed inspection of a specific area, such as heritage sites, rather than the overall survey of infrastructures and buildings.

### 4.3. Standards and Guidelines

Table 1 shows existing standards and guidelines of IRT for delamination detection for infrastructures and buildings. These documents employ the passive analysis scheme and the reflection mode. Target objects include bridge decks [47], concrete structures [79,117,118], and tile and render finish façades [96,118]. The documents describe recommendations or requirements for environmental conditions and IR cameras.

Regarding environmental conditions, four factors are generally stipulated: solar irradiation, ambient temperature, wind, and weather. All documents recommend the survey with direct sunlight exposure because solar irradiation has significant energy input and generates high thermal contrast. ASTM [47] for bridge decks and the Japanese IRT standard [118] for tile or plaster finishes require continuous solar irradiation for 2–3 h before and during the measurement. Additionally, the Japanese Public Work Research Institute [91] defines the minimum intensity of solar irradiation. Regarding nighttime inspection, Japan Society for Non-Destructive Inspection [118] recommends the time window for the survey of 9 p.m. to 5 am, while Japan Building & Equipment Long-Life Cycle Association [70] recommends 2–4 h after sunset. The daily change of ambient temperature is considered as another stimulus. Thus, some documents mentioned recommended values, for example, a daily change of at least 10 °C for shaded areas [96,117]. In terms of wind, low wind speed is considered a suitable condition because wind removes heat from the surface. Thus, several documents stipulate that wind speed is less than 5 or 6.7 m/s [47,96,117]. These wind speeds correspond to 3 “Gentle Breeze” or 4 “Moderate Breeze” in the Beaufort wind force scale [119], respectively. Regarding weather, a fine day is recommended in all the documents since it provides direct sunlight and high daily ambient temperature change. Additionally, some documents [47,96] require no rain for one day and dried surfaces. In summary, long-duration solar irradiation, high daily ambient temperature change, low wind speed, and fine weather are commonly recommended conditions.

IR camera specifications, distance from a target object to an IR camera, and observation angle are also mentioned in the documents. One of the specifications is temperature resolution, represented as noise equivalent temperature difference (NETD) [120]. The NETD indicates a temperature difference that can be distinguished from noise. ASTM [47] requests an IR camera with the NETD of 0.2 °C or less, and other guidelines [117,118] demand that of 0.1 °C or less. With the recent development of IR camera technologies, even affordable cameras can commonly satisfy NETD of 0.1 °C or less [60,121]. Regarding distance, a short distance is preferable due to less infrared attenuation by the atmosphere. However, documents set a wide range of distances, such as 5–20 m [118] and 5–50 m [117]. Concerning angle, the limitations of observation angle vary depending on the documents, such as 30° [96] and 60° [117,118]. Large tolerances about distance and angle may be due to limitations of accessibility and surroundings of infrastructures and buildings.

### 4.4. Comparison with Other NDTs

In addition to IRT, several NDTs were developed to detect delamination on infrastructures and buildings: audio methods, stress wave methods, and electromagnetic methods [17,18,19,122,123].

Audio methods are based on a feature that when a mechanical impact is applied on a target object from outside, delamination areas produce impact sound with a frequency significantly different from intact areas (hollow sound); an inspector listens to the impact sound and evaluates delamination areas. The suitable method of giving mechanical impact relies on target objects. For vertical surfaces such as buildings or tunnel linings, coin tapping testing, which uses coins, steel rods, or lightweight hammers, is widespread [96,122]. For bridge decks, chain dragging testing was standardized by ASTM [124]. The disadvantage of these audio methods is that interpretation depends on inspectors. Thus, a method of analyzing sound with fast Fourier transformation (FFT) to evaluate objectively was proposed [125].

Stress wave methods utilize characteristics of stress-wave propagation in a target object. Among the methods, impact-echo testing (IE) and ultrasonic testing (UT) can detect delamination. IE is a method that a mechanical impact is applied to a target object, then the frequency of the wave reflected on delamination is analyzed with FFT [126,127]. UT is a method in which a transducer emits ultrasonic pulses into a target object. An adjacent transducer receives the pulses reflected on delamination, rebar, or the object’s boundary (pitch-catch method). The travel time of the pulses determines path length [123]. Additionally, a synthetic aperture focusing technique (SAFT) using multiple transducers can image the position and depth of delamination in 3D [126]. Although the stress wave methods require contact with an object, they can measure delamination depth.

Ground penetration radar (GPR) employs electromagnetic pulses [128]. Electromagnetic pulses propagate through a target object from an antenna. A receiver captures the pulses reflected on the boundary between media having different dielectric constants. GPR is widely used to inspect the inside of structures or bridge decks because it can detect delamination, voids, rebars, and buried objects [129,130].

IRT was compared with these NDTs about delamination detection: coin tapping testing [126], chain dragging testing [19,131], IE [19,126,132,133,134], UT [19,126,135], and GPR [19,126,128,129,132,135]. In the case of bridge deck inspection, IRT is as accurate as or slightly less accurate than IE [19,135] and more than as accurate as GPR [19,129]. Additionally, IRT is more suitable for detecting shallow delamination than GPR and UT, while IRT cannot detect deep delamination [126,132,135]. The advantages of IRT are that it can collect data without contact, inspection speed is the fastest among these NDTs, and the inspection cost is relatively low [19,135]. The disadvantage is that IRT is more sensitive to environmental conditions than other NDTs, so that the reliability of IRT is not high [132,135]. Therefore, a method that combines IRT with other NDTs to enhance accuracy, reliability, and measurable depth was investigated [89,128,131,133,134,136].

## 5. Recent Studies of Affecting Factors on IRT for Infrastructures and Buildings

The detectability of IRT is affected by many factors, including environmental conditions, delamination properties, target objects, and IR cameras [52]. Thus, the existing standards and guidelines of IRT state recommended conditions as mentioned in Section 4.3. However, these recommendations are not sufficiently quantitative and explicit. Furthermore, it is not practical for all the surfaces of an infrastructure to meet these recommendations, such as solar irradiation for a long duration. In addition, environments differ depending on the survey region. Therefore, affecting factors and these impacts on detectability were studied. Table 2 covers studies over the last 20 years on these factors using different environmental conditions, delamination properties, target object, and IR camera. This section compiles and discusses experimental methodologies adopted.

### 5.1. Test Method

**Figure 4:**
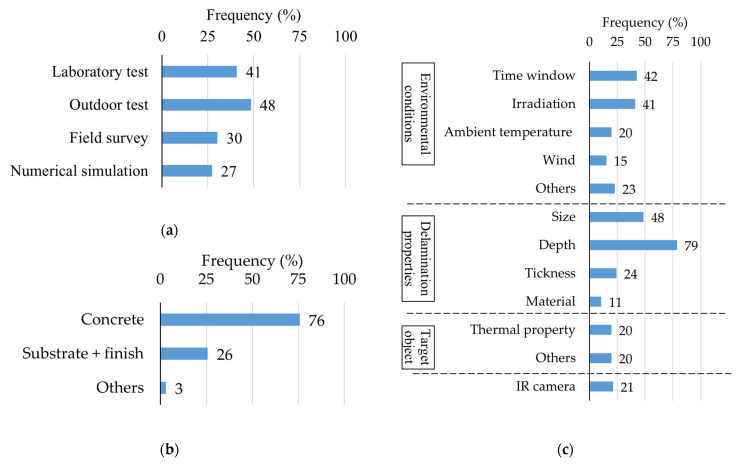
Statistics about methodologies in previous studies: (**a**) distribution of test methods; (**b**) distribution of target objects; (**c**) distribution of study factors.

Test methods used in the previous studies are classified into four categories: laboratory test, outdoor test, field survey, and numerical simulation. Figure 4a indicates the frequencies of test methods employed by 66 studies. Laboratory tests and outdoor tests were mainly used by 41% and 48% of the literature, respectively. In contrast, the frequencies of field surveys and numerical simulations were low at approximately 30%. Thus, laboratory tests and outdoor tests predominated in previous studies.

**Figure 5:**
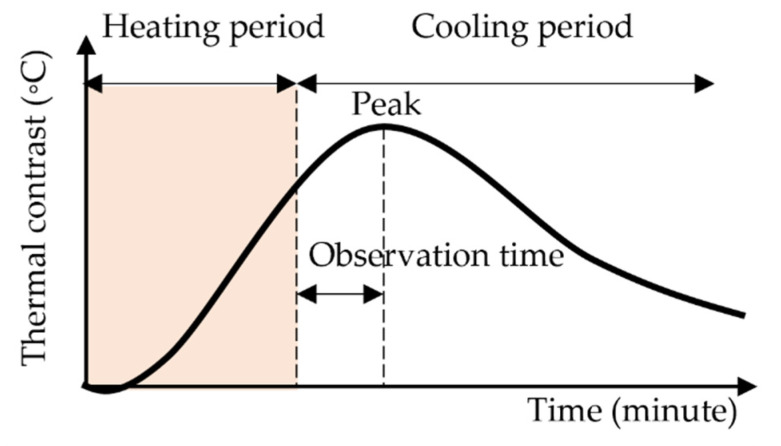
Typical thermal contrast transition in a laboratory test.

In one laboratory test, specimens are prepared with polystyrene foam plates or other Insulation materials embedded to imitate delamination. Figure 5 shows a typical thermal contrast transition in a laboratory test. Artificial lamps heat the surface of a specimen during a heating period of 5–120 min [82,154]; thus, thermal contrast rises. After the lamps are turned off, thermal contrast continues to rise and reaches a peak. Then, thermal contrast decreases. The advantage of laboratory tests is that study factors can be controlled. The tests can investigate each factor independently and IRT detectability in ideal conditions with less noise. Many studies examined the impacts of delamination size and depth on detectability under laboratory conditions [54,82,167]. However, as it is not easy to simulate complex and dynamic outdoor conditions in a laboratory, the test is not appropriate to examine suitable time windows for the survey.

An outdoor test places specimens with simulated delamination in an outdoor location and observes the specimens for several days [159]. Thermal contrast generally behaves the curve shown in Figure 2c. The tests can examine detectability considering the combined effects of environmental factors [103,145,146]. Hence, the tests can investigate suitable time windows for the survey. However, environmental conditions around the specimens greatly depend on test region, climate, surface direction, etc. Thus, the results of outdoor tests are limited to a specific region and are not easy to be generalized.

A field survey is a method of inspecting existing infrastructures or buildings. The survey is often used to verify the results of laboratory tests and outdoor tests [101,148]. The difference from outdoor tests is that a field survey cannot control delamination properties; thus, some studies have compared the results using other NDTs [20,131,160]. Another disadvantage is the influence of noise, for example, reflections from surroundings [175,176], emissivity variation on the surface [27], subsurface material differences [97], and uneven solar heat gain [97]. In addition, thermal contrast can be caused by other subsurface defects, such as water penetration and high moisture content [20,176,177].

Numerical simulation or modeling may provide useful information on the impact of factors such as irradiation [20,170], defect size, and depth [82]. The accuracy of simulation results greatly relies on boundary settings; thus, validation according to laboratory tests or outdoor tests is essential. When modeling outdoor conditions, there are two types of input environmental data: meteorological observatory data [49] and standard environment data [20]. Software packages used in previous studies are general-purpose FEM software (e.g., COMSOL) [88] and transient thermal and humidity movement analysis programs for building envelopes (e.g., WUFI) [148].

### 5.2. Target Object

Figure 4b shows that concrete was used as the target object in 76% of the previous studies because concrete is a fundamental and prevailing material in infrastructures and building structures. On the other hand, the substrate with finishes, the main materials in building facades, was at a low frequency of 26%. Substrates were mainly concrete, but few studies have examined the effects of bricks [162] or stones [54]. Finishes were tiles and mortar renders attached to substrates [109].

### 5.3. Test Location

The results of outdoor tests and field surveys may rely on the test region and surface direction. Most research was conducted under temperate climates, with high daily temperature changes and stable weather, for example, in the US [88] and Europe [20]. In contrast, there are few studies in the tropics [137].

Surface direction is also an important test condition because it relates to the magnitude and time of solar irradiation. In outdoor tests and field surveys, surface directions were mainly horizon or south elevation in the Northern Hemisphere. The horizontal direction assumed bridge decks, and the south elevation is considered ideal conditions with solar irradiation in buildings.

### 5.4. Metric and Criterion

Although metrics and criteria of detectable delamination are critical to identifying delamination and evaluating the impact of factors, there are no unified metrics and criteria. The metrics commonly used in previous studies are thermal contrast and signal-to-noise ratio (SNR).

Thermal contrast or ΔT, the temperature difference between sound and delamination area, is the most commonly used matric because it is simple and easy to analyze. However, the criterion of ΔT to be judged as delamination significantly differ depending on the studies, ranging from 0.2 to 1.2 °C. For example, Hiasa et al. [88,159] and Watase et al. [152] have defined a probable range for detectability as ±0.2 °C or larger and a certain range for detectability as ±0.4 °C or larger in outdoor tests for the concrete specimens. The reason was that Clark et al. [95] reported delamination on concrete bridges and masonry bridges was recognized when ΔT was more than ±0.2–0.3 °C. Additionally, Hiasa et al. [159] stated that ΔT of at least 10–20 times camera’s NETD allowed inspectors to distinguish delamination from thermal noise. On the other hand, several studies [24,56,89,157,168] adopted ΔT of 0.5 °C as the criterion according to ASTM [47] for bridge deck inspection. Moreover, higher ΔT was used as the criterion. Farrag et al. [102] used ΔT of ±0.8 °C due to a more confident assessment. Another value of ΔT was ±1 °C. Washer et al. [100,145,146] mentioned that 1 °C was an order of magnitude larger than the thermal sensitivity of general IR cameras and was twice of ASTM [47]. Similarly, Raja et al. [170] employed ΔT of 1 °C in numerical simulations because the wind effect reduced ΔT to half. Chiang & Guo [158] also suggested ΔT of 1 °C as the criterion according to field surveys for tiled façades.

Meanwhile, some studies proposed multiple criteria of ΔT depending on test methods. For example, Zheng et al. [174] mentioned that it was difficult to identify temperature anomalies correctly by naked eyes when ΔT was less than 0.3 °C in the laboratory test and 1.2 °C in the outdoor test. Moreover, Sultan & Washer [163] examined the criteria quantitatively using receiver operating characteristics (ROC) analysis. As a result, 0.8 °C in the outdoor test and 0.6 °C in the field survey were optimum ΔT to balance true-positive and false-positive rates of delamination areas. As described above, the problem of thermal contrast is that the criterion is not established adequately. The reason may be that environmental conditions change thermal contrast and background noise.

The SNR is utilized as the metric to evaluate the detectability of delamination [10,67,83,85,161] objectively. The *SNR*, which is used in engineering, compares single levels of a target area to signal levels of background noise, calculated by the following equation [85]:

(4) $$SNR\;\left(\mathrm{dB}\right)=20\log_{10}\left(\left|S_{area}-N_{area}\right|/\sigma_{noise}\right),$$

where *S_area_* is the average temperature value in the delamination area, *N_area_* is the average value in the surrounding area, and *σ_noise_* is the standard deviation in the surrounding area. Positive *SNR* means detectable delamination, and negative *SNR* means undetectable. The advantage of the *SNR* is that because of signal level evaluation, the metric and criterion can be applied not only to raw IR images but also processed images, such as PPT or PCT [10,169].

## 6. Affecting Factors of Detectability

This section compares and synthesizes previous studies on factors affecting delamination detectability. Figure 4c shows the frequency of study factors in the previous studies. Respectively, 43% and 40% of the studies examined the effects of time windows and irradiation. Meanwhile, only 20% and 15% of the studies dealt with ambient temperature and wind, respectively. This is probably because radiant heat transfer by sunlight is considered larger than convection heat transfer by the air. Regarding delamination properties, 78% and 48% of the studies investigated the effect of delamination depth and size, respectively. On the other hand, the effects of target objects and IR cameras were studied by approximately 20% of the literature. Therefore, time windows, irradiation, size, and depth are the main factors that attract attention among researchers.

### 6.1. Environmental Conditions

#### 6.1.1. Time Window

Suitable time windows to conduct passive IRT are critical information for getting proper IR images to analyze. Multiple environmental factors, such as irradiation and ambient temperature change, can affect thermal contrast intricately. Thus, time windows are generally examined by outdoor tests and numerical simulations. Table 3 shows suitable time windows and interchange times in each direction under fine weather proposed by the literature.

Regarding horizontal surface and south elevation, available time windows proposed by the literature are generally around 10 a.m. to 3 p.m. due to the presence of solar irradiation [56,90,101,132,147]. However, suitable or optimum time windows vary. Chiang & Guo [158] mentioned recommended time window of 10 a.m. to 12 p.m. according to the field survey for tile façades in Taiwan. Meanwhile, Scott et al. [147] suggested that the recommended time window was 12 p.m. to 3 p.m. for up to 6.5 cm deep delamination because of a time lag between the maximum solar loading at noon and thermal contrast responses. Pozzer et al. [24] statistically analyzed meteorological data and thermal contrast. They predicted favorable time windows from 12 p.m. to 3 p.m. due to high solar radiation, high ambient temperature, and low pressure.

Furthermore, several studies proposed that suitable time windows relied on delamination depth. The reason is that the deeper delamination is, the longer it takes for heat flow to reach delamination. Washer et al. [145] showed that the optimum time for 2.5 cm deep delamination was 5:40 h after sunrise and that for deep delamination of 12.7 cm was 9 h after sunrise. Similarly, Kee et al. [89] reported that 6.4 cm deep delamination could not be detected 3:45 h after sunrise even though it satisfied 3 h of solar irradiation required by ASTM [47]. In contrast, Watase et al. [152] argued that any time was suitable for shallow delamination of 1 cm. Additionally, delamination size can affect time windows. For example, Scott & Kruger [149] stated that the small delamination of 25 cm diameter generated the maximum contrast 4:30 h after sunrise, whereas the large delamination of 50 cm did 6:30 h after sunrise.

Meanwhile, several studies have focused on interchange times, which can not detect delamination due to low thermal contrast. Edis et al. [20] calculated that the interchange times happened on tile façades from 5:30 a.m. to 6:50 a.m. and from 4:30 p.m. to 5:50 p.m. Similarly, Janků et al. [101] confirmed that the times occurred at 8 a.m. and 4 p.m. in the outdoor test. Hiasa et al. [90] also reported that the interchange time windows were 1 h in the morning and 2 h in the evening.

Overall, many studies examined suitable time windows for horizontal surface and south elevation during daytime. Although it is affected by delamination properties, the suitable time window is generally noon to early afternoon when delamination depth is under 6 cm.

Regarding east and west elevation, time windows with solar irradiation on the elevation should be optimal. It means that the suitable time for east elevation is in the morning and that for the west elevation is in the afternoon. For example, Buare et al. [153] observed in the field survey that the maximum contrast appeared at 8:30 a.m. on the east elevation, and thermal contrast declined toward 12:30 p.m. Thus, they proposed that the beginning of sun exposure was the optimal time window. Similarly, Lourenço et al. [162] pointed out that the desirable time was the first 1:30 h after the beginning of solar irradiation on the west elevation. Chiang & Guo [158] also mentioned that the recommended time windows were 9 a.m. to 11 a.m. on the east elevation and 12 p.m. to 2 p.m. on the west elevation. Therefore, suitable time windows for east or west elevation can be after direct sunlight exposure.

Nighttime or the cooling period is a candidate for suitable time windows; however, this is still being debated. One opinion is that nighttime is not appropriate or impossible to conduct IRT. Yehia et al. [132] failed to detect delamination in the outdoor test at night. Additionally, Freitas et al. [148] argued that nighttime inspection was available, while delamination during nighttime was less evident than those during daytime. The opposite opinion is that nighttime is more optimum than daytime because of less noise on IR images [19,90,161] or a long measurable duration [56,90]. Hiasa et al. [90] observed that IR images captured during daytime had much noise caused by nonuniform heating and shadows from surroundings. Moreover, Mac et al. [56] stated that the available time window during nighttime was from 7:30 p.m. to 2 a.m., which was longer than the window during daytime from 10 a.m. to 3 p.m. The difference in the literature on detectability during nighttime is considered due to environmental conditions.

Furthermore, there are still two opinions about suitable time windows during nighttime: early night or early morning. Hiasa et al. [153] mentioned that the maximum negative thermal contrast of 2.5 cm deep delamination occurred at around 7 pm, and the delamination was well recognized. Lourenço et al. [162] also insisted the optimum time was 1 h after the surface was covered in shades for tile facades. On the other hand, Kee et al. [89] suggested that even deep delamination, which was undetectable during daytime, could be detected 45 min after sunrise because of a long cooling duration until early morning. Hence, these studies indicate that optimum time windows during nighttime are dependent on delamination depth.

Shaded areas, soffit, or north elevation, which has no solar irradiation on the inspected surface, may exist on infrastructures and buildings. In these areas, suitable time windows during daytime are generally around noon due to the peak of ambient temperature; however, these time windows are shorter than those of sunny areas [101,103,158]. Regarding daytime and nighttime, previous studies do not agree with which time window is suitable. Rocha et al. [103] argued that thermal contrast during nighttime was smaller than that during daytime. In contrast, Watase et al. [152] proposed that midnight was the favorable time window for deck soffit rather than noon because of a high probability of days when thermal contrast exceeded the criterion of detectability. Thus, further studies are needed on suitable time windows for shaded areas.

As explained above, suitable time windows for IRT proposed by previous studies are not consistent. The reason is that the windows are affected not only by surface direction but also by environmental conditions and delamination properties. Therefore, investigating suitable time windows for each region and the target object is required to conduct IRT properly.

#### 6.1.2. Irradiation

Solar irradiation is a primary stimulus producing heat flow [20,145]. It reaches 700 W/m^2^ on a south elevation and 1300 W/m^2^ on a horizontal surface at noon [88,148,159]. Previous studies have demonstrated that the larger the energy input is, the higher thermal contrast and SNR are generated in laboratory tests [10,85,139,157,169,170]. In contrast, delamination is difficult to be detected under low or no solar irradiation conditions [20,148,162]. In addition, detectable delamination depth is proportional to the heating time in the laboratory test [85,167]. Meanwhile, excessive energy input could decline the thermal contrast of shallow delamination [82,91]. Overall, a large amount of irradiation is generally a preferable condition for IRT.

Few studies quantitatively investigated the relationship between irradiation and thermal contrast. Washer et al. [145] conducted the outdoor tests for three months and argued that the daily total solar loading, not the maximum solar loading, had a high correlation with the maximum thermal contrast. The authors suggested that the total daily solar roading of at least 700 Wh/m^2^ was required for 5.1 cm deep delamination to generate the detectable thermal contrast of 1 °C based on statistical analysis. Likewise, Raja et al. [170] proposed that the total irradiation of 680 Wh/m^2^ produced the thermal contrast of 1 °C for 6.3 cm deep delamination based on the numerical simulations. In addition, the authors stated that a heat flux rate greatly influenced thermal contrast, especially for shallow and small delamination. These studies indicate that total irradiation of approximately 700 Wh/m^2^ could be required to conduct passive IRT.

#### 6.1.3. Ambient Temperature

Daily ambient temperature change is one of the drivers to generate thermal contrast due to convection heat transfer. The daily change is a primary heat source in shaded areas or under cloudy weather [20]. Multiple studies concluded that significant daily ambient temperature change increases thermal contrast and is preferred for IRT based on outdoor test results [100,101,103,137,146,152]. However, the amount of daily change required in shaded areas is not consistent among previous studies. For example, Janků et al. [101] confirmed that the daily change of more than 10 °C was necessary, while Rocha et al. [103] also insisted at least 5.4 °C. Likewise, Washer et al. [100] suggested a daily change of at least 8 °C for 5.1 cm deep delamination. Additionally, the authors proposed that the rate of ambient temperature change of at least 1.5 and −1.7 °C/h was favorable for daytime and nighttime inspection, respectively. Overall, the high daily ambient temperature change is favorable for passive IRT in shaded areas, although the requirement is still debated.

Ambient temperature values might influence thermal contrast. Tran et al. [164] mentioned that high ambient temperature increased thermal contrast, especially for large and shallow delamination, although the effect of temperature was significantly smaller than irradiation.

For buildings, an ambient temperature difference between indoor and outdoor can also affect thermal contrast. Edis et al. [20] conducted parametric studies on the effect of the difference using numerical simulation. The difference enhanced thermal contrast on the surface during daytime when the outdoor temperature was hotter than the indoor temperature. Thus, the effect of the ambient temperature difference should be considered when the difference is more than 10 °C.

#### 6.1.4. Wind

Wind velocity is an environmental factor to be considered when performing passive IRT, as it relates to convection heat transfer [49]. High wind velocity increases heat transfer between the surface and the air [178]. Thus, the wind has different effects on thermal contrast depending on the presence of solar irradiation.

Under the condition of solar irradiation or during the heating cycle, high wind velocity decreases thermal contrast. The reason is that the surface temperature of a target object is generally higher than ambient temperature, so that the wind removes heat energy from the surface. For example, Washer et al. [144] statistically analyzed the relationship between the maximum thermal contrast and average wind velocities in the outdoor tests. As a result, average wind velocity tended to be low when thermal contrast was high. Moreover, Raja et al. [170] quantitatively investigated the effect in the laboratory tests and stated that thermal contrast decreased as the wind velocity increased, especially for deep delamination. For example, the wind velocity of 7 m/s reduced thermal contrast by half for 6.3 cm deep delamination. Furthermore, the authors stated that the slight wind velocity of 1.4 m/s also decreased thermal contrast by 20%. Therefore, low wind velocity is preferable in sunny areas when solar irradiation is used as thermal stimulation.

In contrast, in shaded areas, high wind velocity could increase thermal contrast. The reason is that the surface temperature is generally lower than ambient temperature, and high wind velocity increases energy input from the air to the object’s surface. Washer et al. [100] pointed out that high wind velocity improved thermal contrast based on the outdoor tests. Although high wind velocity is preferable in shaded areas, Washer et al. [146] suggested a guideline that average velocity during 6 h is limited to 4.4 m/s (16 km/h) because high wind velocity might indicate unstable weather conditions. Overall, wind positively affects thermal contrast in shaded areas, as opposed to sunny areas.

#### 6.1.5. Relative Humidity

Relative humidity (RH) is considered to affect thermal contrast due to two theories. One theory is that high RH increases convection heat transfer between the object surface and atmosphere [179]. Thus, in shaded areas or soffit, high RH increases the effect of ambient temperature change on thermal contrast during the heating and cooling cycle [87,172]. The other theory is that high RH increases water adsorption on the surface. Rocha et al. [103] suggested that high RH during nighttime enhanced negative thermal contrast because water adsorption increases moisture content and thermal conductivity near surfaces. Therefore, high RH is typically a preferable condition for IRT.

However, the effect of RH may be limited and not significant. For example, Tran et al. [164] argued that thermal contrast under high RH was more evident than that under low RH for shallow delamination of 1 cm in the laboratory test. In comparison, there was no difference in thermal contrast for 2–3 cm deep delamination. Additionally, Washer et al. [87] mentioned that the effect of RH was not significant in sunny areas because the effect of solar irradiation is dominant. These studies indicate that the positive effects of high RH are less significant than other factors.

#### 6.1.6. Others

Weather is closely related to other environmental factors. A sunny day is optimal for IRT regardless of sunny or shaded areas due to high solar radiation and high daily ambient temperature change [56,101,147,162]. A cloudy day is not recommended because of the small energy input from irradiation and ambient temperature change [148,162]. A partially cloudy day should also be avoided as rapid irradiation changes might make delamination identification difficult [162]. Regarding nighttime, a clear sky is also optimum because radiative cooling removes heat energy from the surface and enhances thermal contrast [90,103]. Overall, fine weather is desirable at all times. However, IRT cannot always be performed under fine weather, so that identifying acceptable weather conditions for IRT is necessary in practice.

A method to predict thermal contrast from meteorological data was proposed. Watase et al. [152] proposed multilinear regression formulas to estimate thermal contrast on bridge deck and soffit under Florida climate conditions. The variables of the formulas were ambient temperature at a bridge and ambient temperature and atmospheric pressure at a nearby meteorological observatory. Furthermore, Washer & Fuchs [180] developed a web-based application to predict whether passive IRT can be carried out based on meteorology records and weather forecasts. Likewise, Pozzer et al. [24] performed multivariate regression analysis under Brazilian climate conditions, considering interactions of meteorological variables. They mentioned that significant dependent variables were ambient temperature, atmospheric pressure, solar radiation, and survey time. In contrast, the size and depth of delamination and wind velocity were not significantly related to thermal contrast. Although these predictions are useful in practice, these formulas are limited to specific regions and are not general.

Seasons are related to the amount of solar irradiation and daily ambient temperature change. Hence, the effects of seasons were examined using numerical simulation, but the results are not consistent. Hiasa et al. [159] concluded that seasonal effects were minor on the horizontal plane in Florida. In contrast, Pozzer [173] mentioned that spring and summer were desirable for IRT in Brazil because of high daily ambient temperature change. Therefore, preferred seasons for IRT depend on the region.

### 6.2. Delamination Properties

#### 6.2.1. Size

Detecting small delamination at the early stage of deterioration leads to ensuring public safety. As shown in Figure 4c, half of the studies have examined the effect of the delamination size. Regarding the relationship between size and thermal contrast, Hiasa et al. [159] showed that size had a much more substantial effect on thermal contrast than thickness and volume of delamination by numerical simulation about outdoor tests. Moreover, Raja et al. [170] argued that the total heat input to create the contrast of 1 °C was inversely proportional to the area; thus, large delamination needed less input heat to be detected. However, Hiasa et al. [88] stated the size effects converged at approximately 40 cm. Additionally, the authors examined the impact of an aspect ratio of the delamination area. The thermal contrast of delamination with an aspect ratio of 25% or more was comparable to the contrast equal to the area of square or circle. In general, large-size delamination with a high aspect ratio has significant thermal contrast and is easily detected.

Delamination size is also related to the response time of the maximum thermal contrast. Maierhofer et al. [139] confirmed that observation time, shown in Figure 5, became longer as the area increased. Similarly, Scott & Kruger [149] mentioned that the delay of the maximum thermal contrast from peak irradiation increased as the size was large in the outdoor test. Thus, delamination size may change optimum time windows for inspection.

#### 6.2.2. Depth

Depth from delamination to the surface significantly affects thermal contrast. Thus, detectable depth is an essential indicator of IRT abilities. Approximately 78% of the studies include depth as study parameters, as shown in Figure 4c. The range of depth examined is wide and depends on target objects assumed in the literature. For buildings, delamination was generally set to a depth of 0.5–3 cm [109,133,137,141,154,162]. For concrete civil infrastructures, the delamination depth was set to a depth of approximately 2–8 cm [82,85,101,102,103,139,157,159,163,166], which are standard concrete cover thickness [181]. Moreover, some studies examined 10 cm or more deep delamination to evaluate IRT limitations [19,82,88,89,102,132,139,145].

It is not easy to detect deep delamination as deep delamination has low thermal contrast. Table 4 lists the maximum detectable depth in previous studies by one-time data analysis. An overall trend is that maximum detectable depth depends on conditions. The detectable depth in (b) and (c) outdoor tests with solar irradiation tends to be deeper than that in (a) laboratory tests. The reason can be the difference in the total amount and time of energy input to test objects.

Furthermore, detectable depth was a controversial and much-disputed subject even under the same test condition. In (b) and (c) outdoor tests with solar irradiation, Washer et al. [87] mentioned that 12.7 cm and 7.6 cm deep delamination were detectable during the heating and cooling cycles, respectively. In contrast, Kee et al. [89] argued that 6.4 cm and 15.2 cm were the maximum detectable depths during the heating and cooling cycles, respectively. Besides, Hiasa et al. [159] reported that 5.1 cm deep delamination was not detectable at any time, and approximately 3 cm was the maximum depth in Florida. Similarly, Mac et al. [56] stated that delamination of up to 4 cm depth could be detected in South Korea. These differences in the detectable depth could be due to differences in environmental conditions, delamination properties, target objects, and metrics.

Depth estimation was also of great interest to researchers because depth is essential information to evaluate severity. For example, AASHTO Guide Manual for Bridge Element Inspection [182] assesses the severity of delamination based on its size and depth. Currently, two approaches to estimate depth were proposed: response time and thermal contrast magnitude.

The estimation method based on response time utilizes that delamination depth correlates with the time from energy input to the generation of thermal distribution on the surface [183]. In laboratory tests, this response time is defined as observation time, a difference from the end of the heating period to the peak [167], as shown in Figure 5. Many studies estimated delamination depth accurately using the observation time [10,82,85,139,157,164,167]. However, the coefficient of estimation formulas changes depending on environmental conditions and the thermal diffusivity of target objects [157,167]. Moreover, delamination size also influences response time and the observation time [139,149]. Thus, the estimation method based on response time is possible only under a specific controlled environmental condition, such as laboratory tests.

The estimation method based on thermal contrast magnitude uses the correlation between thermal contrast and depth. Tran et al. [164] showed in the laboratory test that the inverse of the cube of depth was proportional to the loss of contrast with relatively high accuracy. The authors insisted that this method was practical because it can quickly estimate depth without time-consuming analysis of observation time. Similarly, Raja et al. [170] demonstrated a linear correlation between the square of the depth and the total energy input to generate thermal contrast of 1 °C. However, these methods are difficult to be applied to outdoor tests because environmental conditions are not constant and change dynamically. Hence, Hiasa et al. [88] proposed a method of comparing actual thermal contrast to calculated thermal contrast at each depth by numerical simulation. Although it can estimate depth in outdoor tests, the method requires obtaining time-series data of irradiation and ambient temperature and the thermal properties of the target object. In addition, numerical simulation must be conducted for each depth based on those data.

As described above, depth estimation methods using response time or thermal contrast magnitude are possible under constant or controlled conditions such as laboratory tests. However, since environmental conditions fluctuate, further research is needed to estimate depth under outdoor conditions.

#### 6.2.3. Width to Depth Ratio

**Figure 6:**
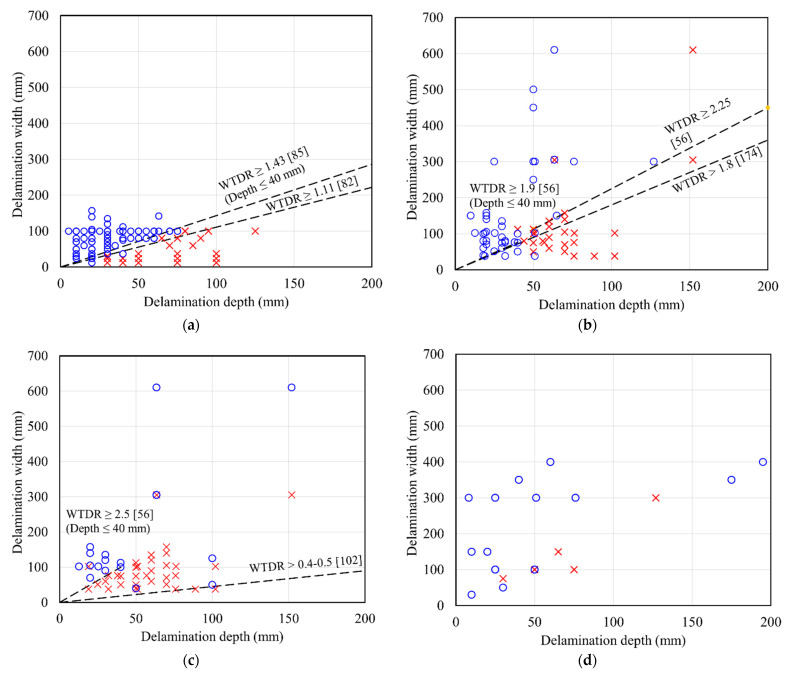
Synthesises of literature about detectability with respect to depth and size of delamination in concrete: (**a**) laboratory test; (**b**) outdoor test with solar irradiation measured during heating cycle (daytime); (**c**) outdoor test with solar irradiation measured during cooling cycle; (**d**) outdoor test in shaded areas. Legends are that blue circles indicate detectable delamination, while red crosses indicate undetectable delamination. Synthesized data have following terms: target object is ordinary concrete; analysis method is one-time data analysis; width represents diameter or shortest side of delamination; detectability is determined according to each study. Sources are [10,56,82,85,87,89,100,101,102,103,132,133,143,145,146,147,149,157,159,164,167,170,174,186].

Delamination width and depth are closely related to detectability while interacting. It is generally considered that the minimum detectable width is at least 1–2 times the depth or more [184]. Thus, many studies have investigated the width-to-depth ratio (WTDR) criterion of detectable delamination in laboratory tests [10,82,85,157,167] and outdoor tests [56,102,174]. Figure 6 shows the syntheses of the previous results of detectability with respect to the width and depth of delamination in concrete specimens. The data were categorized according to test conditions. The WTDR criteria proposed by the literature are also displayed in Figure 6. A WTDR corresponds to the slope of the straight line through the origin of figures. The overall tendency is that the upper left region of each graph, high WTDR, clearly has a high probability of delamination detection. The reason can be that the larger WTDR delamination is, the higher the thermal contrast is and the easier it is to detect by IRT [56,174,185]. Furthermore, the results of the same width and depth delamination are not consistent enough, especially for delamination near the proposed WTDR criteria. This inconsistency can be due to the difference in environmental conditions, delamination properties, and detection metrics.

More specifically, each condition has a different tendency for detectable delamination distribution and WTDR criteria. In (a) laboratory tests, the distribution results are almost consistent among the literature compared to outdoor tests. This is probably because such laboratory tests can optimize energy input and remove unintended noise from the surroundings. Additionally, the detectable and undetectable regions are relatively separated by a straight line. Thus, the WTDR criteria proposed by the literature are relatively low values of 1.11–1.43 [82,85]. This means that laboratory tests can detect small and deep delamination. In (b) outdoor tests with solar irradiation measured during the heating cycle, the WTDR criteria of 1.8–2.25 were proposed [56,174], which are higher than those in (a) laboratory tests. In (c) outdoor tests with solar irradiation measured during the cooling cycle, the distribution of detectable delamination and the proposed WTDR criteria differ significantly depending on the studies. Mac et al. [56] suggested that the WTDR criterion was 2.5 in Korea, whereas Farrag et al. [102] proposed the that of 0.4–0.5 in the UAE. This difference can be attributed to intense solar irradiation during daytime in the UAE. Figure 6c indicates that the proposed WTDR of 2.5 [56] relatively agrees with the results of other studies. In (d) outdoor tests in shaded areas, WTDR criteria were not proposed by previous studies to our knowledge. Although the number of results is not adequate, Figure 6d suggests that the distribution is not significantly different from (c) outdoor tests with solar irradiation measured during the cooling cycle.

As described above, the WTDR criteria of detectable delamination are influenced by test methods, the presence of solar irradiation, measurement cycle, and test regions. As a result of integrating previous studies, WTDR criteria are approximately 1.25 in (a) laboratory test, 2.0 in (b) outdoor test with irradiation during the heating cycle, and 2.5 in (c) outdoor test with irradiation during the cooling cycle and (d) outdoor test in shaded areas.

#### 6.2.4. Thickness

Delamination thickness is also a factor to consider for its impact on detectability. Previous studies have generally set the thickness of 0.1–2 cm by adjusting the thickness of embedded materials. Thick delamination has a low overall heat transfer coefficient; thus, it generates significant thermal contrast regardless of environmental conditions or measurement cycles [20,88,89,102,103,141,146,159]. For example, Kee et al. [89] reported that delamination of 0.1–0.2 cm thickness at 6.4 cm depth was detectable, while thin delamination of 0.03 cm thickness was undetectable. However, the effect of thickness may converge at a certain value. Hiasa et al. [88] showed convergence at 1 cm thickness by the numerical simulation.

Meanwhile, thickness is considered the minor effect on thermal contrast among the geometric factors of delamination [91]. Hiasa et al. [159] demonstrated that the most influential factor was the area of delamination, followed by thickness. Similarly, Farrag et al. [102] showed that thickness was the geometric aspect with the least effect on thermal contrast. These results indicate that IRT is relatively robust to the effect of delamination thickness.

#### 6.2.5. Material

Delamination is usually filled with air; thus, the thermal properties of delamination are considered to resemble the air. However, making air-filled delamination in a concrete specimen with a predetermined size and depth is not easy, except for delamination beneath tiles. To simulate delamination, materials with low thermal conductivity are embedded in specimens. Thus, several studies have examined the effect of embedded materials [54,82,102,132,141,168]. For example, Yehia et al. [132] maintained that air-filled delamination was more visible than delamination simulated with polyethylene foam. Contrary to this, Cotič et al. [82] mentioned no significant difference between thermal contrasts above polystyrene foam and air-filled void. Although the results of these studies are not consistent enough, polyethylene foam is generally used as the material to simulate delamination. The reason may be that the difference between the thermal conductivity of polyethylene foam (0.033–0.045 W/mK [187]) and air (0.022 W/mK) is negligible for that of concrete (1.6–2.1 W/mK [139,157]). Therefore, the results of IRT by polyethylene foam could be applied to the actual delamination.

### 6.3. Target Object

#### 6.3.1. Thermal Property

The materials of the target object affect thermal contrast because heat flow is determined by thermophysical properties of the materials: thermal conductivity, specific heat capacity, and density. The properties of concrete change depending on compression strength and mix proportions. For example, Rocha et al. [103] and Farrag et al. [102] stated that concrete with a low water-to-cement ratio or high strength concrete generated high thermal contrast in outdoor tests because of high thermal conductivity and high density. On the other hand, Maierhofer et al. [143] mentioned that thermal contrast decreased slightly along with the concrete strength increase. Additionally, the authors showed that density significantly affected thermal contrast, while thermal conductivity had minor effects. As mentioned above, there are some debates about the effects of materials on thermal contrast.

Building walls are generally layered with different materials rather than the single material of concrete. When finish materials are the same, substrate materials can also affect thermal contrast. Lourenço et al. [162] examined an external thermal insulating composite system (ETICS) and brick masonry with tile finish. In addition, Meola [141] investigated marble, brick, and tuff with render finish. These studies indicate that subsurface materials with high thermal conductivity generate high thermal contrast. The reason can be that substrate material with high conductivity increase the ratio of the difference in thermal transmission coefficients between sound area and delamination area. This means that delamination becomes difficult to be detected in the order of concrete, bricks and insulation in substrates.

#### 6.3.2. Others

Other factors related to target objects investigated by the previous studies include rebars, water penetration, and surface conditions. The effects of these factors might be inevitable when inspecting existing infrastructures and buildings.

Rebars are usually embedded in concrete parallel to the surface to reinforce concrete structures. Rebars have a high thermal conductivity of 12.5 W/mK, much higher than concrete of 1.8 W/mK. Therefore, rebars may diffuse heat flow parallel to the surface, resulting in low thermal contrast. According to laboratory tests and outdoor tests, the effect of rebars is different depending on the relative position of rebars and delamination [85,102,143,147,167]. When delamination occurs between rebars and the surface, rebars have little impact on thermal contrast and detectability [85,167]. On the other hand, when delamination occurs deeper than rebars, the effect is not consistent enough between previous studies. Scott et al. [147] stated no differences in thermal contrast between the presence and absence of rebars. In contrast, Huh et al. [85] argued that delamination indicated significantly lower SNR than delamination above rebars; thus, the delamination under rebars was not easy to be detected. Moreover, rebar density also affects thermal contrast. Maierhofer et al. [143] pointed out that high rebar density slightly decreased thermal contrast. In addition, rebars can influence response time. Tran et al. [167] revealed that rebars above delamination shortened observation time; thus, the depth of delamination may be estimated to be shallower than the actual depth. Overall, the effects of rebars on thermal contrast rely on the relative position between delamination and rebars.

Water penetration or high moisture content in a target object generates nonuniform temperature distribution on a surface due to three physical phenomena: evaporative cooling [25,162,177], the increase in specific heat capacity of the object [175,188], and the increase in thermal conductivity of the object [7,26]. Water penetration may occur at the same time as delamination in target objects. Edis et al. [188] surveyed glazed tile façade buildings and stated that both delamination and high moisture content areas had positive thermal contrast in midafternoon (e.g., 4:30 pm) under sunlight exposure conditions. However, water penetration into a delamination cavity may cause negative effects on thermal contrast and detectability. Lourenço et al. [162] conducted the outdoor tests in which water was poured into the back of nonadhesive tiles. Water penetration created opposite thermal behavior to delamination and decreased thermal contrast. Similarly, Güray et al. [166] stated that water-filled delamination could not be detected at any time. To address the issue caused by water penetration, Lourenço et al. [162] proposed inspecting target objects in different conditions: after rainy days and under dry conditions. Therefore, since water penetration could generate thermal contrast or reduce detectability, IRT surveys after rain or under wet conditions should be avoided.

Surface conditions, such as color and obstacles on the surface, affect IR images. Building facades are generally colored with paint or colorful materials. Lourenço et al. [162] studied the effects of surface color using white and black tiles. Black color, which absorbs a large amount of solar irradiation, contributed to high thermal contrast during the heating and cooling cycle. Thus, surface color affects detectability in sunny areas, and dark colors are advantageous for IRT.

The surfaces of infrastructures and buildings are not always clean and may have small obstacles. Hiasa et al. [90] stated that the obstacles could be discriminated on IR images because obstacles were smaller than a deck surface and quickly heated up and cooled down. The authors also suggested that visual images could help to distinguish obstacles certainly. To complement the information of IR images with visual images, simultaneously capturing IR and visual images is recommended.

### 6.4. IR Camera

#### 6.4.1. IR Camera Type

Two types of IR cameras are generally used for IRT: a short-wavelength (SW) camera and a long-wavelength (LW) camera [79]. Table 5 indicates the characteristics of types of IR cameras. SW and LW cameras can detect infrared rays in the high atmospheric transmission band of 3–5 μm and 8–14 μm, respectively, known as atmospheric windows [189]. This difference in the band creates the characteristics of these cameras.

SW cameras use a cooled quantum detector sensitive to high-energy emissions from hot objects [79]. Thus, the quality of IR images is high when a target object is at a high temperature. In contrast, SW cameras are not suitable for measurements at a low temperature below 10 °C [96]. Additionally, the cameras are less affected by reflections of surrounding buildings or the sky on glazed facades [96]. The disadvantage is that the cameras are susceptible to solar reflections on the surface. Therefore, SW cameras tend to be used at night [156].

LW cameras use an uncooled microbolometer detector sensitive to low-energy emissions. Thus, the quality of IR images is relatively high when a target is at a low temperature. In addition, LW cameras are less subject to solar reflections on surfaces. In contrast, the cameras are susceptible to reflections of surrounding buildings and the sky on glazed tiles or smooth surfaces [14,96,97,160]. Hence, LW cameras are often used for daytime measurements [156]. Currently, many LW cameras are being developed, including affordable models [160] and lightweight models for unmanned aerial vehicles [56,190].

Regarding the influence of IR camera type and model, Hiasa et al. [156,159,160] compared two LW cameras and an SW camera, and Bauer et al. [14] examined two LW cameras of different manufacturers. Although different IR cameras output different temperature values even for the same object, there were no significant differences in thermal contrast and detectability. Therefore, selecting the type of IR camera is advisable according to the type of surrounding noise.

#### 6.4.2. Distance and Spatial Resolution

A short distance from the IR camera to the target object is considered ideal [27,137,160,162]; however, surveys at short distances are not always possible due to the limited accessibility of existing infrastructures. The distance can affect detectability in three aspects: atmospheric attenuation, captured area, and spatial resolution.

Atmospheric attenuation is a phenomenon in which water vapor and carbon dioxide in the atmosphere absorb IR [191]. Due to the low impact of atmospheric attenuation, short distance measurements can provide accurate temperature values with few errors [27]. Furthermore, the effect of distance on detectability depends on the camera types because the atmospheric attenuation relies on spectral ranges [191]. For example, Hiasa et al. [160] mentioned that LW cameras were relatively affected by distance, while SW cameras were less affected. However, both cameras appropriately captured thermal contrasts, which are important to detect delamination. Overall, it is considered that distances within 10 m have little impact on detectability [137,177,192].

The size of a captured area may influence the efficiency of IRT surveys and detectability. The area captured is determined by an IR camera’s field of view (FOV) and distance. FOV indicates the largest area that an IR camera can capture, described in horizontal and vertical degrees, and is determined by the focal length and the detector size of the IR camera. A long-distance measurement can capture a large area at once and improve efficiency. However, this IR image tends to include surroundings or nontarget objects with high or low temperatures. IR cameras automatically adjust the span of the temperature color scale based on the maximum and minimum temperature in an IR image. Therefore, Lourenço et al. [162] stated that the surroundings and nontarget objects widened the scale of the image, making it difficult to emphasize the slight thermal contrast of delamination in visual analysis. Thus, short-distance measurement is recommended.

To measure the temperature value of a small area accurately, at least a smaller spatial resolution than the area is required [79]. Spatial resolution refers to the physical size of a target object per pixel and is determined by the multiplication of instantaneous field of view (IFOV) and distance. IFOV is determined by FOV and sensor resolution (the number of pixels). Therefore, spatial resolution becomes large as the distance increases and the sensor resolution decreases. Hiasa et al. [160] mentioned that the IR camera with a small spatial resolution (sensor resolution is 640 × 512 pixels) had higher sensitivity for detecting delamination than that with a large spatial resolution (sensor resolution is 320 × 240 pixels) at the same distance. Thus, using the IR camera with high sensor resolution is one way to keep detectability for long-distance measurements. However, the sensor resolution of IR cameras is lower than that of visual cameras and is commonly limited to 640 × 512 pixels [55]. Hence, Scott et al. [147] suggested using a telescope lens of small IFOV for long-distance measurements to keep the spatial resolution. Selecting an appropriate distance, FOV, and sensor resolution is important for detecting small delamination.

As described above, distance is related to detectability from the aspects of atmospheric attenuation, captured area, and spatial resolution. It is desirable to capture IR images as close as possible while balancing productivity and limitation of accessibility.

#### 6.4.3. Angle

An observation angle could affect temperature values measured by IR cameras and detectability. This is because the emissivity of objects relies on the angle with respect to the surface. In general, the emissivity of nonmetallic materials is stable from the angles of 0° to 45° and decreases at higher angles [193,194]. Several studies suggested that thermal contrast is stable when angles are within 45°, and delamination can be detected although measured temperature values might change [147,156,190]. Additionally, Ortiz et al. [190] noted that the angle of 0° should be avoided for glazed surfaces because an IR camera may capture the reflection of the inspector or the IR camera on the surface.

At angles above 45°, the detectability of IRT may decline because of thermal contrast reduction or reflection noise. Scott et al. [147] reported that only shallow delamination, which was high thermal contrast, could be detected at the angle of 80° in the outdoor test. Moreover, Ortiz et al. [190] argued that measurement errors increased sharply due to reflections from the sky and the sun. Although the survey with angles above 45° may detect delamination, the angle within 45° is desirable to keep the reliability of IRT.

#### 6.4.4. Platform

When surveying a wide area, mounting an IR camera on a platform can enhance the IRT survey’s efficiency compared to by hand. For example, in the bridge deck inspection, an IR camera fixed to the top of a car continuously captures a road lane [195]. However, IR images captured on vehicles may be blurry or low quality due to the effects of moving speed or vibration. Thus, ASTM [47] limits the speed to 16 km/h or less. To survey with normal car speed without closing road lanes, Hiasa et al. [160,161] have examined the effects of speed on IR images using the two types of IR cameras. As a result, the SW camera with fast shutter speed could acquire IR images with high quality at 48 or 64 km/h, whereas the LW cameras with slow shutter speed captured blur IR images. Hence, measurement at high-speed movement requires SW cameras.

Recent developments in robotics allow inspectors to use unmanned aerial vehicles (UAVs) as a platform to inspect infrastructures and buildings [64,196,197]. UAVs with IR cameras can access any location without scaffolds and efficiently capture IR images at appropriate distances and angles [165,198,199]. Some studies have compared UAVs with traditional platforms, a tripod or cart, in outdoor tests using LW cameras [56,155]. As a result, mounting the camera on UAVs has little effect on the quality of IR images at a resting state or slow speeds.

## 7. Conclusions

Capturing latent defects at the early stage of delamination even before delaminated objects falling is essential for integral components of infrastructures and buildings. With this in mind, a comprehensive review on the use of IRT to detect delamination on infrastructures and buildings was presented.

Three classifications of IRT for assessing defects were explained to clarify the methodologies used in delamination detection. Regarding delamination detection, the principle, evaluation protocols with one-time and time-series data analysis, and standards and guidelines were consolidated. Additionally, the performance of IRT in detecting delamination was compared with that of other NDTs.

Experimental methodologies employed by studies over the last 20 years on factors affecting delamination detection were discussed. Furthermore, the impact of factors on detectability was also investigated. Factors studied include environmental conditions, delamination properties, target objects, and IR cameras. Although the results of the studies were not always consistent due to the differences in experimental conditions, general desirable conditions for IRT are summarized below:

- Suitable time windows for the inspection depend on the direction of the inspection surface and delamination depth. For shallow delamination on a horizontal surface or south elevation, the windows are noon to early afternoon and late evening to early night.
- A large amount of total solar irradiation is desirable because irradiation is the primary heat source to generate thermal contrast.
- High daily ambient temperature change allows IRT even in shaded areas.
- A low wind velocity is preferable in sunny areas.
- Fine weather is optimum for the heating and cooling cycles because of solar irradiation, high daily ambient temperature changes, and radiative cooling.
- Delamination of large size has high thermal contrast and is easy to detect.
- The detectable depth of delamination is greatly affected by environmental conditions. Delamination of at least 3–5 cm or less could be detected in outdoor conditions.
- The width to depth ratio (WDTR) of delamination also affects detectability. The WTDR criteria of detectable delamination are 1.25 under laboratory conditions and 2–2.5 under outdoor conditions.
- The target object with high thermal conductivity has high thermal contrast, and the detectability is low on the insulation walls or low-strength concrete.
- Water penetration into delamination causes the opposite behavior of the thermal contrast of delamination.
- Dark color surfaces in sunny areas are advantageous for inspection.
- The influence of obstacles on the surface can be removed by complementing IR images with visual images.
- Both types of SW cameras and LW cameras can be used for inspection. An appropriate type should be selected according to the noise of the surrounding environment.
- The close distance from an IR camera to a target object is desirable in terms of atmospheric attenuation, captured area, and spatial resolution while balancing productivity and limitation of accessibility.
- When IR camera platforms, such as vehicles or UAVs, move quickly, SW cameras can collect clear IR images compared with LW cameras.

The results of this study could be used as the benchmarks for setting standardized testing criteria, as well as for comparison of results for future works on the use of infrared thermography for detection of delamination on infrastructures and buildings.

## Figures and Tables

**Table 1:** Existing standards and guidelines of infrared thermography (IRT) for delamination detection and recommended/required environmental conditions.

Document	Target Object	Recommended/Required Environmental Conditions			
		Solar Irradiation	Ambient Temperature	Wind	Weather
ASTM D47888-03 [47]	Bridge deck	A minimum direct solar irradiation for 3 h	An air temperature rise of 11 °C with 4 h of sun for concrete in winter An air temperature rise of 11 °C with 6 h of sun for asphalt in winter	Wind speed of less than 15 mph (6.7 m/s)	Dry for at least 24 h before the survey
Japan Public Work Research Institute [117]	Concrete infrastructure	A minimum direct solar irradiation of 350 Wh/h for 2–3 h	Daily temperature change of more than 10 °C in shaded areas Not suitable for 3–4 h after the maximum or minimum air temperatures	Wind speed of less than 5 m/s	Fine weather
British Instiute of Non-Destructive Testing [79]	Structural finishes	Strong solar exposure		Low wind speed	Fine weather
Japanese Society for Non-Destructive Inspection [118]	Concrete infrastructure, Tile façade, Shotcrete	A minimum direct solar exposure for 2 h			Fine or partly cloudy weather
Japan Building and Equipment Long-Life Cycle Association [96]	Tile façade, Render façade	Around the period of maximum solar irradiation on each elevation 2–4 h after sunset	Daily temperature change of more than 10 °C for shaded elevations	Wind speed of less than 5 m/s	No rain from one day before

**Table 2:** Recent studies on affecting factors of delamination detectability of IRT for infrastructures and buildings.

Author	Year	Test Method	Target Object	Test Location (Outdoor Test or Field Survey)		Study Factors											
						Environmental Conditions					Delamination Properties				Target Object		IR Camera
				Region	Direction	Time window	Irradiation	Ambient Temperature	Wind	Others	Size * (cm)	Depth (cm)	Thickness (cm)	Material	Thermal Property	Others	
Chew [137]	1998	Laboratory test, Outdoor test	Concrete + Tile	Singapore	Vertical		✓	✓	✓		1–15	1	1–1.5				✓
Maierhofer et al. [138]	2002	Laboratory test, Numerical simulation	Concrete, Concrete + CFRP				✓				10–20	1–10	10				
Clark et al. [95]	2003	Field survey	Concrete, Stonemasonry	UK	Vertical, Soffit						No detail				✓		
Maierhofer et al. [139]	2004	Laboratory test, Numerical simulation	Concrete				✓				10–20	1–10	10		✓		
Maierhofer et al. [140]	2005	Laboratory test, Numerical simulation	Concrete				✓				10–20	1–10	10		✓		
Meola et al. [141]	2005	Laboratory test, Field survey	Brick/Marble/Tuff + Render	Italy	Vertical						4–10	1–5.5	0.1–0.2	✓	✓		
Maierhofer et al. [142]	2006	Laboratory test, Numerical simulation, Field survey	Concrete, Concrete + CFRP/Stone, Asphalt,	Germany	Horizon						10–20	2–8	10		✓	Rebar	
Meola [54]	2007	Laboratory test	Brick/Marble/Tuff + Render, Concrete								2–10	1–1.5	0.1–3	✓	✓	Water	
Maierhofer et al. [143]	2007	Laboratory test, Numerical simulation	Concrete								10	6–10	5	✓	✓	Concrete age, Rebar	
Yehia et al. [132]	2007	Outdoor test	Concrete	USA	Horizon						3.8–10.2	1.9–10.2	1.3–5.1	✓			
Cheng et al. [133]	2008	Laboratory test	Concrete, Concrete + Tile								5–16	0.5–3	7–9.5				
Washer et al. [144]	2009	Outdoor test	Concrete	USA	South	✓	✓		✓		30	2.5–12.7	1.3				
Washer et al. [145]	2010	Outdoor test	Concrete	USA	South	✓	✓				30	2.5–12.7	1.3				
Washer et al. [146]	2010	Outdoor test, Field survey	Concrete	USA	North			✓	✓		30	2.5–12.7	1.3				
Gucunski [19]	2012	Outdoor test	Concrete	USA	Horizon	✓					30–61	6.4–15.2	0.03–0.2				
Kee et al. [89]	2012	Outdoor test	Concrete	USA	Horizon	✓					30–61	6.4–15.2	0.03–0.2				
Scott et al. [147]	2012	Outdoor test	Concrete	South Africa	North	✓	✓		✓		15–40	1–6.5	1			Rebar	✓
Edis et al. [97]	2013	Field survey	Tile finish	Portugal	Vertical		✓			Reflection	No detail					Color, Texture, Moisture	✓
Washer et al. [100]	2013	Outdoor test, Field survey	Concrete	USA	North, Soffit			✓	✓		30	2.5–12.7	1.3				
Freitas et al. [148]	2014	Laboratory test, Field survey, Numerical simulation	Concrete + Render	Portugal	South	✓	✓			Weather	No detail						
Rumbayan & Washer [49]	2014	Numerical simulation	Concrete	USA	South, North		✓	✓	✓		30	2.5–12.7	1.3				
Scott & Kruger [149]	2014	Outdoor test	Concrete	South Africa	North	✓					25–52	5–10	5				
Alfredo-Cruz et al. [150]	2015	Outdoor test	Concrete	Colombia	Horizon	✓					15	2.5–7.5	1				
Bauer et al. [14]	2015	Laboratory test	Concrete + Render								No detail						✓
Cotič et al. [82]	2015	Laboratory test, Numerical simulation	Concrete								1.2–10	0.5–12.5	0.5	✓			
Edis et al. [20]	2015	Field survey, Numerical simulation	Brick + Tile	Portugal	Vertical	✓	✓	✓		Season	10	1	1–2				
Edis et al. [111]	2015	Field survey	Brick + Tile	Portugal	South, West	✓					No detail					Moisture content	
Khan et al. [151]	2015	Laboratory test, Numerical simulation	Concrete masonry				✓				20–142	No detail				Size	
Lai et al. [109]	2015	Outdoor test	Concrete + Tile/Render	Hong Kong	East		✓				7.5	0.3–2	0.3–2				
Vaghefi et al. [131]	2015	Field survey	Concrete	USA	Horizon						No detail	5.1–7.9	No detail				
Watase et al. [152]	2015	Outdoor test	Concrete	USA	Horizon, Soffit	✓		✓	✓	Relative humidity, Pressure	10	1–3	0.1				
Bauer et al. [153]	2016	Laboratory test, Field survey	Concrete + Tile	Brazil	East	✓					4	0.4–0.8	0.2				
Bauer et al. [154]	2016	Laboratory test	Concrete, Concrete + Tile				✓				4	0.4–0.8	0.2				
Ellenberg et al. [155]	2016	Outdoor test	Concrete	USA	Horizon						30–61	6.4–15.2	No detail				✓
Farrag et al. [102]	2016	Outdoor test	Concrete	UAE	Horizon		✓	✓	✓	Season	1.2–12.5	2.5–12.5	1.2–5.0	✓	✓	Rebar	
Hiasa et al. [156]	2016	Laboratory test	Concrete								10	1–3	0.1				✓
Huh et al. [157]	2016	Laboratory test	Concrete				✓				3–10	1–3	1				
Chiang & Guo [158]	2017	Field survey	Concrete + Tile	Taiwan	East, West, South, North	✓					No detail						
Hiasa et al. [88]	2017	Outdoor test, Field survey, Numerical simulation	Concrete	USA	Horizon						5–90	1.3–10.2	0.1–10				✓
Hiasa et al. [88]	2017	Outdoor test, Field survey, Numerical simulation	Concrete	USA	Horizon						5–90	1.3–10.2	0.1–10				✓
Hiasa et al. [159]	2017	Outdoor test, Numerical simulation	Concrete	USA	Horizon					Season	10–30	1.3–7.6	0.1–10				✓
Hiasa et al. [160]	2017	Field survey	Concrete	USA	Horizon	✓					No detail						✓
Hiasa et al. [161]	2017	Outdoor test	Concrete	USA	Horizon	✓					10.2	1.3–7.6	0.32				✓
Janků et al. [101]	2017	Outdoor test, Field survey	Concrete	Czech	SouthwestShaded area	✓	✓	✓		Weather	No detail	1–4	No detail				
Milovanović et al. [112]	2017	Laboratory test	Concrete								3–15	1–7	1–4		✓	Concrete age, Rebar	
Lourenço et al. [162]	2017	Outdoor test	IEICS / Brick + Tile	Portugal	West	✓				Weather	30	0.82	0.3		✓	Color, Water penetration	✓
Sultan & Washer [163]	2017	Outdoor test, Field survey	Concrete	USA	Horizon						15.2–60.9	5	2.54				
Tran et al. [164]	2017	Laboratory test	Concrete				✓	✓		Relative humidity	3–10	1–3	1				
Escobar-Wolf et al. [165]	2018	Laboratory test, Field survey	Concrete	USA	Horizon						2.5–10.2	2.5–5	1				✓
Güray & Birgül et al. [166]	2018	Numerical simulation	Concrete		Horizon	✓					10	1.1–4.1	0.2			Water penetration	
Hiasa et al. [90]	2018	Outdoor test, Numerical simulation	Concrete	USA	Horizon	✓				Weather	10	1.3–2.5	0.3			Surface obstacle	
Huh et al. [85]	2018	Laboratory test	Concrete				✓				10	1–8	1			Rebar	
Moropoulou et al. [43]	2018	Laboratory test, Numerical simulation	Stone				✓				1–3	2.5–3.5	No detail		✓		
Rocha et al. [103]	2018	Outdoor test	Concrete	Brazil	Horizon, Shaded area	✓		✓		Relative humidity, Weather	10	2.5–7.5	0.3–1.2		✓		
Tran et al. [167]	2018	Laboratory test	Concrete				✓				7–15	2–8	1			Rebar	
Al Gharawi et al. [116]	2019	Outdoor test	Concrete	USA	South, North		✓			Month	30	2.5–12.7	1.3				✓
Cheng et al. [94]	2019	Laboratory test, Outdoor test, Numerical simulation	Concrete	USA	Horizon	✓	✓				5.1–15.2	3.8–8.9	0.4				
Mac et al. [56]	2019	Outdoor test	Concrete	Korea	Horizon	✓				Weather	5–15.8	2–7	1				✓
Vyas et al. [168]	2019	Outdoor test	Asphalt	India	Horizon	✓					60	5–10	No detail	✓			
Cheng & Shen [110]	2019	Outdoor test, Field test	Concrete	USA	Horizon	✓					25	4.4–9.5	0.4				
Milovanovic et al. [169]	2020	Laboratory test	Concrete				✓				3–10	1–5	1–4				
Pozzer et al. [24]	2020	Outdoor test	Concrete	Brazil	Horizon	✓	✓	✓	✓	Relative humidity, Pressure	5–15	1–5	3				
Raja et al. [170]	2020	Laboratory test, Numerical simulation	Concrete				✓		✓		7–17	2.5–6.3	0.5				
Cheng & Shen [171]	2021	Laboratory test, Outdoor test	Concrete	USA	Horizon	✓	✓	✓			3–6	2.5–10	1–2				
Mac et al. [172]	2021	Outdoor test	Concrete	Korea	Soffit	✓		✓		Relative humidity	35–40	4–19.5	1				
Pozzer et al. [173]	2021	Outdoor test, Numerical simulation	Concrete	Brazil	Horizon	✓				Season	5–15	2–5	3				
Zheng et al. [174]	2021	Laboratory testOutdoor test	Concrete	China	Horizon						4–10	1.8–5	2.4–6.2				

Green shaded cells indicate factors studied by literature. * Size indicates the short side or the diameter of delamination.

**Table 3:** Suitable time windows and interchange times proposed by previous studies.

Direction	Author	Year	Time Windows
Horizontal surface	Yehia et al. [132]	2007	Defects of up to 3.8 cm deep can be detected between 10 a.m. and 3 p.m. Any defects cannot be detected during cooling cycle.
	Gucunski et al. [19]	2012	Defects at 40 min after sunrise are more apparent than at noon.
	Kee et al. [89]	2012	IR images obtained during cooling cycle are more evident than those obtained during heating cycle. Defects cannot be detected 3:45 h after sunrise. Shallow defects of 6.4 cm can be detected 7 h after sunrise.
	Watase et al. [152]	2015	Any time of day is suitable for 1 cm deep delamination, and 6 a.m. is best time.
	Hiasa et al. [90]	2018	Defects can be detected between 10 a.m. and 3 p.m. Defects can be detected between 5 p.m. and 8 am, and maximum contrast appears at 7 p.m. Cooling cycle is more suitable than the heating cycle for the inspection.
	Güray et al. [166]	2018	Favorable time window is between 3 p.m. and 7 p.m.
	Mac et al. [56]	2019	Optimal time windows for up to 4 cm deep defects are between 10 a.m. and 3 p.m. and between 7:30 p.m. and 2:00 a.m.
	Vyas et al. [168]	2019	Interchange times for asphalt unbonded by sand are between 8 a.m. and 10 a.m. and between 2:30 p.m. and 3:30 p.m.
	Pozzer et al. [24]	2020	Ideal time window is between 12 p.m. and 3 p.m.
South elevation (in the Northern Hemisphere)	Washer et al. [144]	2009	Optimum time is from 5–9 h after sunrise.
	Washer et al. [145]	2010	Optimum time is after 5:40 h after sunrise for 2.5 cm deep delamination and 9 h after for 12.7 cm.
	Scott et al. [147]	2012	Recommended time window is between 12 a.m. and 3 p.m. for under 6.5 cm deep delamination.
	Scott & Kruger [149]	2014	Optimum time window is between 11 a.m. and 1 p.m. for under 5 cm deep defects.
	Edis et al. [20]	2015	Interchange times occur between 5:30 a.m. and 6:50 a.m. and between 4:30 p.m. and 5:50 p.m.
	Chiang & Guo [158]	2017	Available time window is between 10 a.m. and 12 p.m.
	Janků et al. [101]	2017	Best time is around noon. Interchange time occurs at 4 p.m.
	Freitas et al. [148]	2018	Best time window is during hours of exposure to sunlight. Defects are less evident during cooling cycle than heating cycle.
East elevation	Bauer et al. [153]	2016	Defects are better visualized in early morning and late afternoon. Interchange time is around 12:30 p.m.
	Chiang & Guo [158]	2017	Available time window is between 9 a.m. and 11 a.m.
West elevation	Chiang & Guo [158]	2017	Available time window is between 12 p.m. and 2 p.m.
	Lourenço et al. [162]	2017	Desirable time during heating cycle is first 1:30 h after beginning of irradiation exposure. Desirable time during cooling cycle is beginning of cycle or 1 h after beginning of shadowing.
Shaded area/Soffit/North elevation (in the Northern Hemisphere)	Watase et al. [152]	2015	Favorable time window is midnight.
	Chiang & Guo [158]	2017	Available time window time is between 11 a.m. and 1 p.m.
	Janků et al. [101]	2017	Best conditions occur around noon.
	Rocha et al. [103]	2018	Best time window is between 10 a.m. 2 pm, specifically at noon. Interchange times are around 7 a.m. and 5 p.m.
	Mac et al. [172]	2021	First optimal time window is 7 h after decks are exposed to sunlight until 0.5 h after decks are not exposed. Second one is from 1.5 h to 3.5 h after decks are exposed to sunlight. Third one is 8 h after decks are not exposed to sunlight until 1 h after decks are exposed.

**Table 4:** Maximum detectable depth in literature.

Conditions	Maximum Detectable Depth in Literature
(a) Laboratory test	6 cm [143], 7 cm [10,85,167], 7.5 cm [82], 10 cm [138]
(b) Outdoor test with solar irradiation measured during heating cycle (daytime)	3 cm [159], 3.2 cm [174], 4 cm [56,101], 5.1 cm [88,132], 6.5 cm [147], 7.5cm [150],10 cm [102], 12.7 cm [87,144]
(c) Outdoor test with solar irradiation measured during cooling cycle (nighttime)	3 cm [159], 4 cm [56,101] 10.2 cm [88], 12.5 cm [102], 12.7 cm [87], 15.2 cm [89]
(d) Outdoor test in shaded areas	4 cm [101] 5 cm [103], 7.6 cm [87], 19.5 cm [172]

**Table 5:** Characteristics of types of IR camera.

Items	Short-Wavelength (SW) Camera	Long-Wavelength (LW) Camera
Spectral range	3–5 μm	8–14 μm
Detector type	InSb, Quantum detector	Microbolometer, Thermal detector
Cooling	Cooling	Uncooling
Thermal sensitivity, NETD	Fine	Middle
Shutter speed	Fast (e.g., 10 μs–10 ms)	Slow (e.g., 10 ms)
Camera cost	High	Low–middle

## Data Availability

Not applicable.
